# Uncovering the Antibacterial Potential of a Peptide-Rich Extract of Edible Bird’s Nest against *Staphylococcus aureus*

**DOI:** 10.4014/jmb.2402.02052

**Published:** 2024-07-12

**Authors:** Thi-Phuong Nguyen, Tang Van Duong, Thai Quang Le, Khoa Thi Nguyen

**Affiliations:** 1 NTT Hi-Tech Institute, Nguyen Tat Thanh University, Ho Chi Minh City 700000, Vietnam; 2 Vietnam National Museum of Nature, Vietnam Academy of Science and Technology, Hanoi 100000, Vietnam

**Keywords:** Antibacterial, edible bird’s nest, ethanol, peptide, *Staphylococcus aureus*

## Abstract

The diverse pharmacological properties of edible bird’s nest (EBN) have been elucidated in recent years; however, investigations into its antibacterial effects are still limited. In the present study, we explored the antibacterial activity of a peptide-rich extract of EBN against *Staphylococcus aureus*, a notorious pathogen. The EBN extract (EEE) was prepared by soaking EBN in 80% ethanol for 2 days at 60°C. Biochemical analyses showed that peptides at the molecular weight range of 1.7-10 kDa were the major biochemical compounds in the EEE. The extract exhibited strong inhibition against *S. aureus* at a minimum inhibitory concentration (MIC) of 125 μg/ml and a minimum bactericidal concentration (MBC) of 250 μg/ml. This activity could be attributed to the impact of the extract on cell membrane integrity and potential, biofilm formation, and reactive oxidative species (ROS) production. Notably, the expression of biofilm- and ROS-associated genes, including *intercellular adhesion A* (*icaA*), *icaB*, *icaC*, *icaD*, and *superoxide dismutase A* (*sodA*), were deregulated in *S. aureus* upon the extract treatment. Our findings indicate a noteworthy pharmacological activity of EBN that could have potential application in the control of *S. aureus*.

## Introduction

Edible bird’s nest (EBN), a salivary product of *Aerodramus* and *Collocalia* swiftlets, has a long record of use as a health delicacy in Asian countries. It consists of proteins (~60%), carbohydrates (~9% sialic acid, 7.2%galactosamine, 5.3% glucosamine, 16.9% galactose, and 0.7% fucose), and inorganic salts (~2%) [1, 2]. The popular EBN extracts, prepared by boiling method or enzymatic hydrolysis, confer noteworthy health benefits. These include enhancement of immunity, alleviation of memory loss, promotion of cell proliferation, as well as antiviral, anti-inflammatory, antioxidant, anti-tyrosinase, neuroprotective, and anti-aging effects [3]. Sialic acid has been elucidated as the main component in the EBN extracts responsible for the inhibition of influenza virus infection and tyrosinase activity [4, 5]. Meanwhile, proteins are other active ingredients participating in the antioxidant and cell proliferative abilities of the EBN extracts [6, 7].

The antibacterial capacity of EBN was previously investigated; however, the obtained results were dependent on the type of EBN extract. In the studies by Hun *et al*. and Babji *et al*., both the aqueous and alcalase-digested extracts of EBN did not inhibit gram-negative (*Escherichia coli* and *Klebsiella pneumoniae*) and gram-positive (*Staphylococcus aureus*) bacterial strains [8, 9]. On the other hand, a study by Saengkrajang *et al*. showed the inhibitory effect of the methanol and ethyl acetate extracts of EBN on *E. coli* and *S. aureus*. The distinct behavior of the methanol and ethyl acetate extracts might be rooted in the enrichment of antibacterial compounds by these two solvents [10].

Although neither the underlying bioactive constituents nor the mechanism of action of the methanol and ethyl acetate extracts have been characterized, the study of Saengkrajang *et al*. suggests the antibacterial potential of EBN and inspires us to gain insight into this property. In our present work, ethanol was employed for EBN extraction due to its non-toxic, easy-to-handle nature and its suitability for extracting antimicrobial peptides [11, 12]. The experiments were performed with *S. aureus*, which was previously used by Saengkrajang *et al*. [10]. Moreover, as *S. aureus* has developed strong drug resistance and is one of the most formidable pathogens causing mortal infections, a natural extract with antibacterial activity against *S. aureus* is of great interest in the pharmaceutical industry [13]. Our findings revealed the antibacterial mechanism and possible bioactive compounds of EBN against *S. aureus* and also offer a possible therapeutic approach for management of this pathogen.

## Materials and Methods

### Preparation of the Peptide-Rich Extract of Edible Bird’s Nest

Processed house EBN (20 g) harvested in Binh Thuan Province, Vietnam, was ground into powder with a mortar and pestle and then submerged in 80% ethanol (1 L) for 2 days at 60°C. The extract was evaporated in a rotary evaporator (Labtech, Republic of Korea) and freeze-dried in a freeze dryer (Operon, Republic of Korea). The freeze-drying step was performed until a constant weight of the dried extract was obtained to ensure the complete removal of water and ethanol. Subsequently, 30% dimethyl sulfoxide (DMSO, Merck, Germany) was used to dissolve the extract (designated here as the EEE) to a final concentration of ~10 mg/ml. The absence of ethanol in the EEE was confirmed by the negative result in the triiodomethane (iodoform) test [14].

### Analysis of Protein/Peptide Content and Qualitative Determination of Alkaloids, Phenolics, Flavonoids, and Terpenoids in the Extract

Proteins/peptides in the EEE were analyzed by sodium dodecyl sulfate–polyacrylamide gel electrophoresis (SDS-PAGE). A Bradford assay was used to measure the concentration of proteins/peptides as previously described [15].

The presence of alkaloids, phenolics, and terpenoids in the EEE was qualitatively determined according to previous descriptions [16-18]. Briefly, alkaloids were detected by Bouchadard’s and Dragendorff ’s reagents. Ferric chloride and lead tetraacetic acid were employed to assess the presence of phenolic compounds. For terpenoids, Salkowski's test was used in the qualitative assay.

### Determination of the Minimum Inhibition Concentration (MIC) and Minimum Bactericidal Concentration (MBC) of the Extract

The MIC of the EEE on *S. aureus* (ATCC 25293) was determined by a dilution method using resazurin as a colorimetric indicator for metabolically active cells [19]. *S. aureus* at the cell density of 5 × 10^6^ CFU/ml was aliquoted into a 96-well plate. The bacteria cells in Mueller Hinton broth (MHB, Himedia, India) were treated with various concentrations of the EEE (31.25, 62.5, 125, 250, 500, 1000, and 2000 μg/ml) and DMSO as a negative control. The plate was incubated at 37°C for 24 h, followed by the addition of 0.1% resazurin and further incubation at 37°C for 2 h. The color change from pink (the color of resorufin, a reduced product of resazurin) to blue (the color of resazurin) indicates the inhibition of bacterial growth. The MIC was considered as the lowest concentration of the extract that inhibits the visible growth of bacteria.

To determine the MBC of the extract, different concentrations of the EEE (1×MIC, 2×MIC, 4×MIC, and 8×MIC) were employed for the assay. After being treated with the EEE for 24 h, *S. aureus* cells were spread out onto a Mueller Hinton agar (MHA, Himedia) plate and incubated at 37°C for 24 h. The MBC was determined as the lowest concentration of the extract at which the cells were threatened with extinction and colonies failed to develop on the MHA plate.

### Assessment of the Growth of *S. aureus*

To observe the antibacterial activity of EEE against *S. aureus* in a concentration- and time-dependent manner, the bacterial cells at a density of 5 × 10^6^ CFU/ml were exposed to the EEE (1/8×MIC, 1/4×MIC, 1/2×MIC, 1×MIC, 2×MIC, and 4×MIC) and DMSO (negative control) for 0, 1, 2, 4, 8, 12, and 16 h. The bacterial turbidity was measured at 600 nm in a microplate reader (ELx800, BioTek, Winooski, VT, USA). Additionally, a drop of treated bacterial cells (10 μl) was spread onto an MHA plate and the formation of colonies on the plate was used to assess the bactericidal effect of the EEE.

### Assessment of the Cell Membrane Integrity

The cell membrane integrity of *S. aureus* was determined based on the release of cell contents (nucleic acids and proteins) into the medium [20]. *S. aureus* cells were incubated with the EEE (1/8×MIC, 1/4×MIC, and 1/2×MIC) at 37°C for 4 h. The suspensions were then centrifuged at 13,000 ×*g* for 5 min to obtain the supernatant. The concentrations of released proteins and nucleic acids were measured using a Nanodrop One^C^ (Qiagen, Germany) at 260 nm and 280 nm, respectively.

### Assessment of the Cell Membrane Potential

The membrane proton motive force assay was conducted using the membrane potential-sensitive fluorescent probe bis(1,3 dibutylbarbituric acid) trimethine oxonol (DiBAC4(3), Merck) [21]. *S. aureus* cells at the density of 5×10^6^ CFU/ml were treated with different concentrations of the EEE (1/8×MIC, 1/4×MIC, and 1/2×MIC) and DMSO (negative control) for 4 h. Subsequently, treated cells were washed three times with 20 mM of glucose in HEPES buffer (5 mM, pH 7.2). The bacterial cells at optical density 0.5 at 600 nm were incubated with 1 mM of DiBAC4(3) at 37°C in the dark. The fluorescence intensities were monitored in black polystyrene plates every 10 min for 60 min in total at an excitation wavelength of 492 nm and an emission wavelength of 518 nm in a Victor NIVO 3F Microplate Reader (Perkin Elmer, USA).

### Assessment of Biofilm Formation

The assay was performed by crystal violet staining as previously described with a slight modification [22]. Briefly, 5 × 10^6^ CFU/ml of *S. aureus* cells cultured in MHB medium were treated with various concentrations of the EEE (1/8×MIC, 1/4×MIC, and 1/2×MIC) for 4 h at 37°C. The bacterial turbidity was recorded at 600 nm in a microplate reader (ELx800, BioTek). The supernatants were discarded, and the biofilms were gently washed three times with phosphate saline buffer (PBS) to deplete planktonic cells. The biofilms were then stained with 1%crystal violet (Merck) for 20 min, followed by a step of washing the stain with a mixture of ethanol and acetic acid (50:50, v/v). The absorbance of the samples was measured at 590 nm and normalized with the bacterial turbidity.

### Assessment of Reactive Oxygen Species (ROS) Production

The assay used 2’,7’-dichlorofluorescein diacetate (DCFH-DA, Merck) to measure the production of ROS as previously described [23]. Three different concentrations of the EEE (1/8×MIC, 1/4×MIC, and 1/2×MIC) were added to the medium containing *S. aureus* at the cell density of 5 × 10^6^ CFU/ml, followed by incubation at 37°C for 4 h. The supernatant obtained by centrifugation at 4°C for 10 min at 1,000 ×*g* was treated with 100 mM of DCFH-DA for 1 h. The level of released ROS was measured at an excitation wavelength of 485 nm and an emission wavelength of 530 nm in a Victor NIVO 3F Microplate Reader (Perkin Elmer).

### Field Emission Scanning Electron Microscopy (FE-SEM)

*S. aureus* cells were incubated in MHB medium at 37°C for 6 h and then treated with the EEE (1×MIC, 2×MIC, and 4×MIC) or DMSO (negative control). After further incubation at 37°C for 4 h, the cell suspensions were centrifuged at 70 ×*g* for 10 min and the supernatant was discarded. The obtained pellets were washed twice with 0.1 M PBS (pH 7.2) and fixed with 2.5% (v/v) glutaraldehyde in 0.1 M PBS overnight at 4°C. Next, the cells were dehydrated by sequential concentrations of ethanol (30, 50, 70, 80, and 100% v/v) and submerged in bis(trimethylsilyl)amine (HMDS, Merck). The bacterial cells were then dried in a desiccator and platinum-covered by cathodic coating. The images were taken under an FE-SEM microscope (S4800, Hitachi, Japan).

### Quantification of Gene Expression

*S. aureus* cells exposed to 1/8×MIC, 1/4×MIC, and 1/2×MIC of the EEE or DMSO (negative control) for 4 h were processed for total RNA extraction using an RNeasy Kit (New England Biolabs, USA). The quality and concentration of RNA samples were verified by using a Nanodrop One^C^ spectrophotometer (Qiagen) and gel electrophoresis (2% agarose gel).

cDNAs were synthesized using a LunaScript RT SuperMix Kit (New England Biolabs). The mRNA expression of the genes involved in the biofilm formation (*intercellular adhesion A* (*icaA*), *icaB*, *icaC*, and *icaD*) and ROS content (*superoxide dismutase A* (*sodA*)) were assessed using Luna Universal qPCR Master Mix (New England Biolabs). Filamenting temperature- sensitive mutant Z (*ftsZ*) was used as a reference gene for normalization by the delta threshold cycle method [24]. Primer sequences (5’→3’) in the qPCR analysis are as follows: *icaA*-F: TTGTCGACGTTGGCTACTGGGATA and *icaA*-R: TGGAACCAACATCCAACACATGGC, *icaB*-F: AGCAGT CACTCCGAACTCCAATGA and *icaB*-R: TCATGGAATCCGTCCCATCTCT, *icaC*-F: GTCCTATTAGGTCAA TGGTATGGCT and *icaC*-R: TAGCACGGTATCGTGAAACGCTGT, *icaD*-F: GGTCAAGCCCAGACAGAG GGAATA and *icaD*-R: AGACACAAGATATAGCGATAAGTGCT, *sodA*-F: TTCTGGGAGTTACTTTCACCAAA and *sodA*-R: CTGCTTTGTCAGCAAATTCTTTT, *ftsZ*-F: ATCCAAATCGGTGAAAAATTAACAC and *ftsZ*-R: CCATGTCTGCACCTTGGATTG.

### Statistical Analysis

All assays were conducted in three independent experiments. One-way analysis of variance (ANOVA) was employed for the statistical comparison among samples in the assays of cell contents, biofilm, and ROS. Student’s *t*-test was used in the analysis of gene expression. The statistical differences were considered significant as *p* ≤ 0.05.

## Results

### Contents of Protein/Peptide, Phenolics, Terpenoids, and Alkaloids in the EBN Extract

As shown in the SDS-PAGE electropherogram, there was a protein band with the molecular weight of more than 250 kDa and a thick peptide smear at the molecular weight range of 1.7-10 kDa that predominated in the gel (Fig. S1). Further estimation of protein/peptide content by Bradford assay determined an equivalent concentration to the initial concentration of the EEE (~10 mg/ml). Additionally, all the tests that qualitatively scanned for phenolic compounds, terpenoids, and alkaloids showed undetectable or ignorable levels of these compounds in the EEE. Our data imply that peptide is a major ingredient of the EEE with the extraction efficacy of 0.25%. Thus, the EEE was herein considered a peptide-rich extract.

### Antibacterial Activity of the Extract Against *S. aureus*

**Fig. 1:**
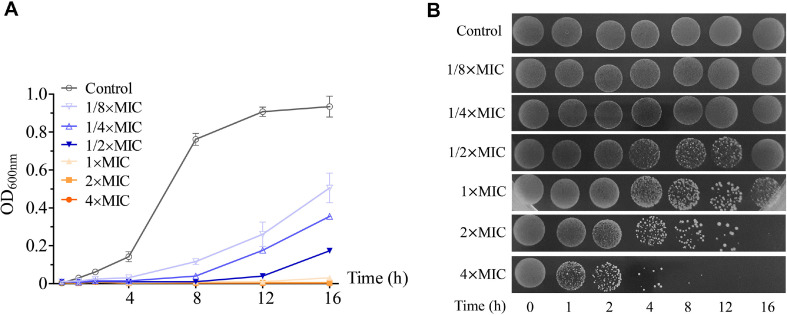
The EEE inhibited the growth of *S. aureus*. (**A**) Measurement of optical density at 600 nm (OD_600nm_). (**B**) Images of colony formation on the agar plate. *S. aureus* was treated with DMSO (control), 1/8×MIC, 1/4×MIC, 1/2×MIC, 1×MIC, 2×MIC, and 4×MIC of the extract for 0, 1, 2, 4, 8, 12, and 16 h. Each data point was presented as mean ± SD of three independent experiments.

In our assay, the EEE exhibited inhibitory activity against *S. aureus* (MIC = 125 μg/ml) and this inhibition was time- and concentration-dependent. While the bacterial growth in the control was seen after 1 h of incubation at 37°C, it was halted in the 1×MIC-, 2×MIC-, and 4×MIC-treated samples at 16 h of incubation. In the samples exposed to 1/8×MIC, 1/4×MIC, and 1/2×MIC of the EEE, no growth of *S. aureus* cells was obtained during 4 h, 8 h, and 12 h, respectively, suggesting a reduced growth rate of the bacteria under these sub-MIC concentrations (Fig. 1A and 1B). Our result is similar to that previously observed for the *Mannheimia haemolytica* strain 55518 under sub-MIC concentrations of chlortetracycline [25]. The altered growth kinetics at sub-MIC concentrations might be due to the impairment of physiological and biochemical functions associated with the bacterial growth [26]. On the agar plate assay, no bacterial colonies appeared in the samples treated with 2×MIC for 16 h and 4×MIC for 12 h, indicating the bactericidal effect of the EEE on *S. aureus* (MBC = 250 μg/ml) (Fig. 1B).

### Effect of the Extract on the Morphology of *S. aureus* Under FE-SEM

**Fig. 2:**
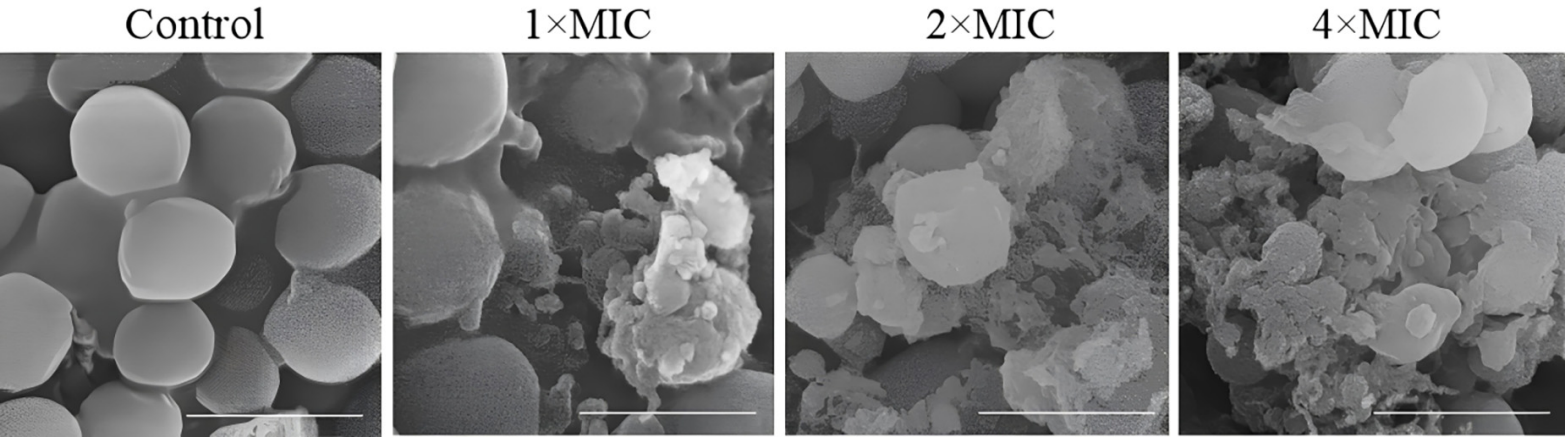
The EEE altered the morphology of *S. aureus* cells. FE-SEM images of bacterial cells treated with DMSO (control), 1×MIC, 2×MIC, and 4×MIC of the extract for 4 h. Scale bar: 1 μm.

According to our observations, the EEE at the concentrations of 1×MIC, 2×MIC, and 4×MIC caused noticeable changes in the morphology of *S. aureus*. Control cells exhibited a typical spherical shape, whereas some of the bacterial cells exploded when exposed to the EEE at 1×MIC for 4 h. The cells were more severely damaged with the extension of the concentrations of the EEE (2×MIC and 4×MIC) (Fig. 2).

### Effect of the Extract on the Cell Membrane Integrity and Potential of *S. aureus*

**Fig. 3:**
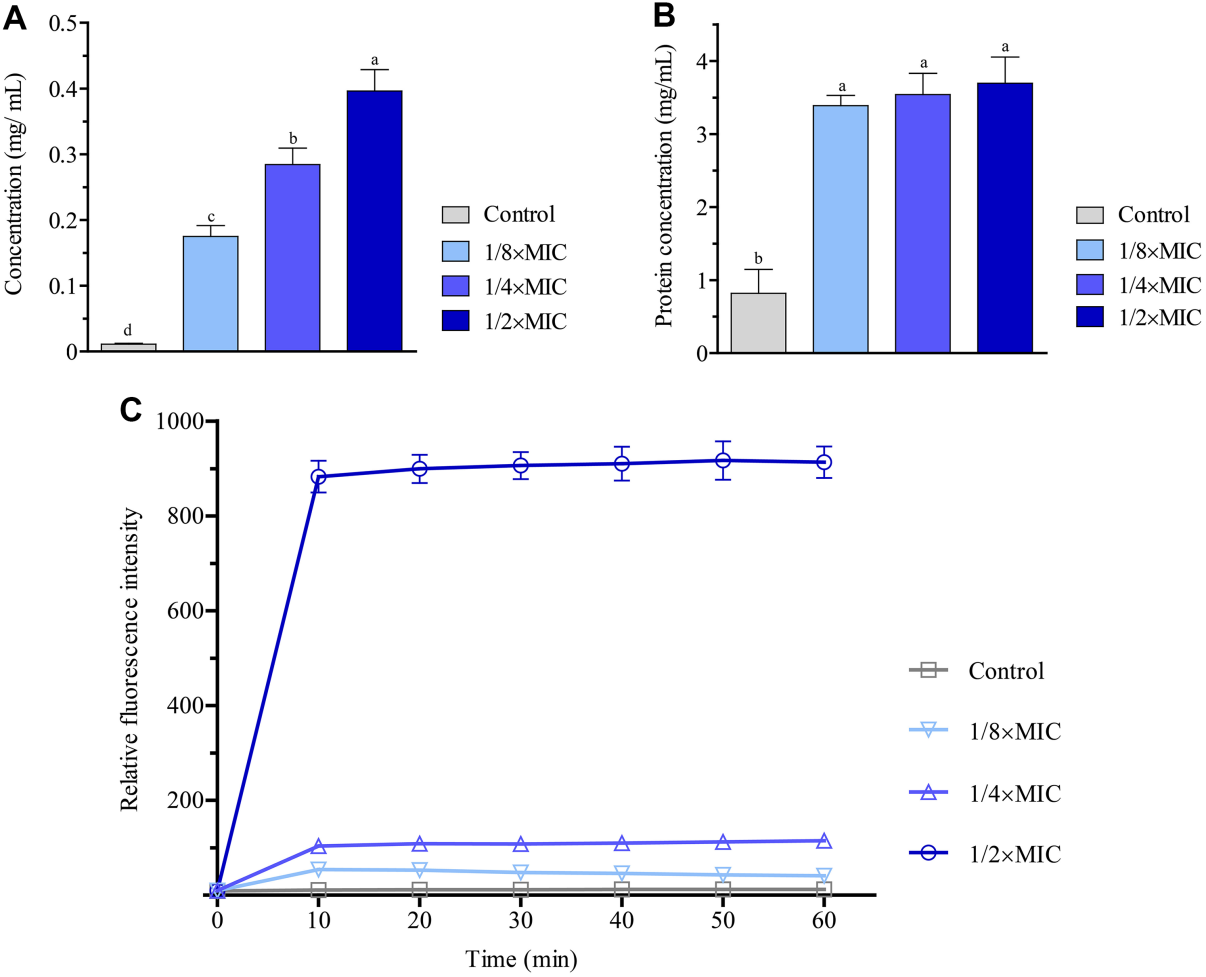
The EEE impaired cell membrane integrity and potential of *S. aureus*. (**A**) Quantification of nucleic acid concentration released into the medium. (**B**) Quantification of protein concentration released into the medium. (**C**) Quantification of fluorescence intensity of DiBAC4(3). *S. aureus* treated with DMSO (control), 1/8×MIC, 1/4×MIC, and 1/ 2×MIC of the extract for 4 h. The fluorescence intensities were calculated relative to the level of the sample at 0 h. Each data bar or point was presented as mean ± SD of three independent experiments. Statistical comparisons among samples were performed by ANOVA. Different letters indicate statistical differences with *p* ≤0.05.

The leakage of intracellular constituents such as nucleic acids and proteins into the medium can be associated with cell membrane permeability and is considered an index for bacterial cell membrane integrity [20, 27]. Upon the addition of the EEE, remarkable amounts of nucleic acids measured by the absorbance at 260 nm were released into the medium in a dose-dependent manner. The highest value of leaked nucleic acids was achieved in the sample exposed to 1/2×MIC for 4 h, which is approximately 45-fold higher than that of the control (Fig. 3A). Similarly, an increase of 4 folds in the protein concentration was observed in all EEE-treated samples (Fig. 3B). These results show the ability of the EEE to destruct the membrane integrity of *S. aureus* cells.

Changes in the cell membrane potential can considerably affect cellular energetics and signal transductions [28]. Thus, we assessed the impact of the EEE on the cell membrane potential of *S. aureus* using a voltage-sensitive fluorescence probe, DiBAC4(3). When the cell membrane is depolarized, the fluorescent dye enters the cell membrane and enhances the fluorescence intensity [29]. In our assay, compared to the control, the samples treated with the EEE for 4 h, particularly at the concentration of 1/2×MIC, boosted the fluorescence intensity of the probe (Fig. 3C). This finding suggests impairment of the cell membrane depolarization, and consequently the cell membrane potential of *S. aureus* by the extract.

### Effect of the Extract on Biofilm Formation

**Fig. 4:**
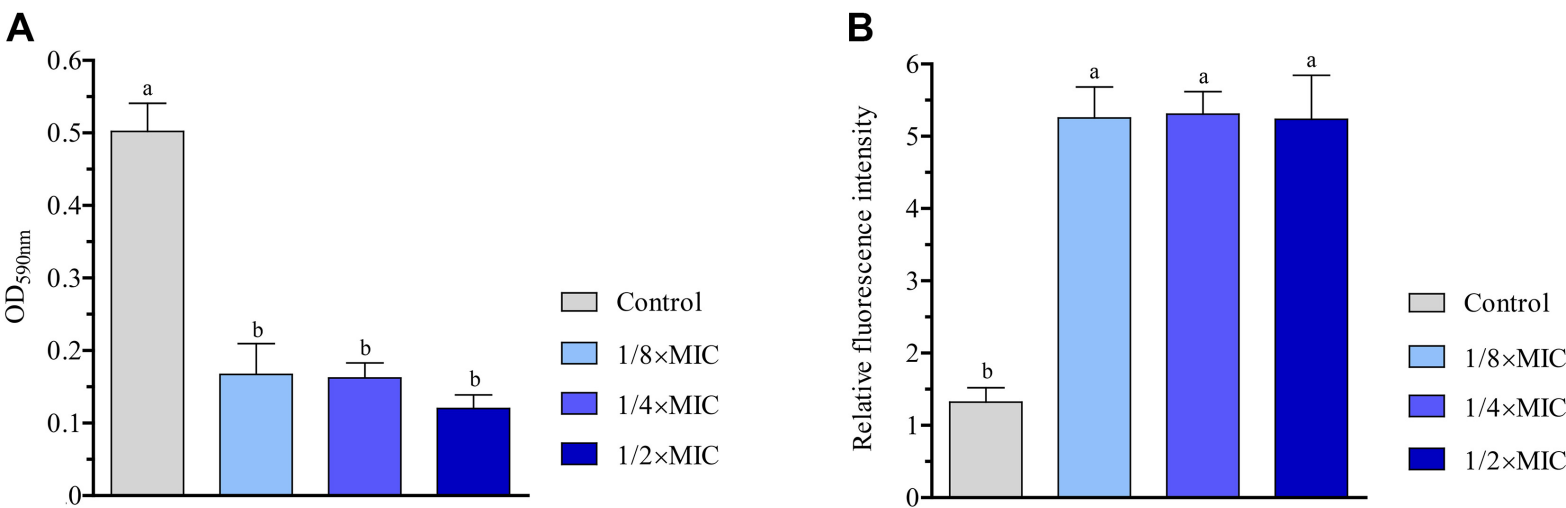
The EEE decreased biofilm formation and increased ROS content in *S. aureus*. (**A**) Quantification of biofilm content by optical density at 590 nm (OD_590nm_). (**B**) Quantification of fluorescence intensity of H2-DCFDA. Bacterial cells were treated with DMSO (control), 1/8×MIC, 1/4×MIC, and 1/2×MIC of the extract for 4 h. The fluorescence intensities were calculated relative to the level of the sample at 0 h. Each data bar was presented as mean ± SD of three independent experiments. Statistical comparisons among samples were performed by ANOVA. Different letters indicate statistical differences with *p* ≤ 0.05.

Bacterial biofilm composed of bacterial cells in a self-produced extracellular polymeric matrix is an important strategy used by bacteria to survive under oligotrophic environments [30]. Here, we determined if the antibacterial activity of the EEE against *S. aureus* was also involved in the biofilm formation. In our analysis, 4 h of treatment resulted in a 2.5-fold decrease in biofilm production irrespective of the extract concentrations, demonstrating the anti-biofilm potential of the EEE on *S. aureus* (Fig. 4A).

### Effect of the Extract on ROS Content

ROS are deadly weapons damaging bacterial cells via the induction of oxidative stress [31]. To investigate the association of the EEE with ROS, we measured the ROS level using a well-known fluorescent dye, H2-DCFDA. As seen in Fig. 4B, the extract elevated the ROS level nearly 5-folds, after only 4 h of treatment, and this tendency was independent of the extract concentrations used in the assay.

### Effect of the Extract on Gene Expression

**Fig. 5:**
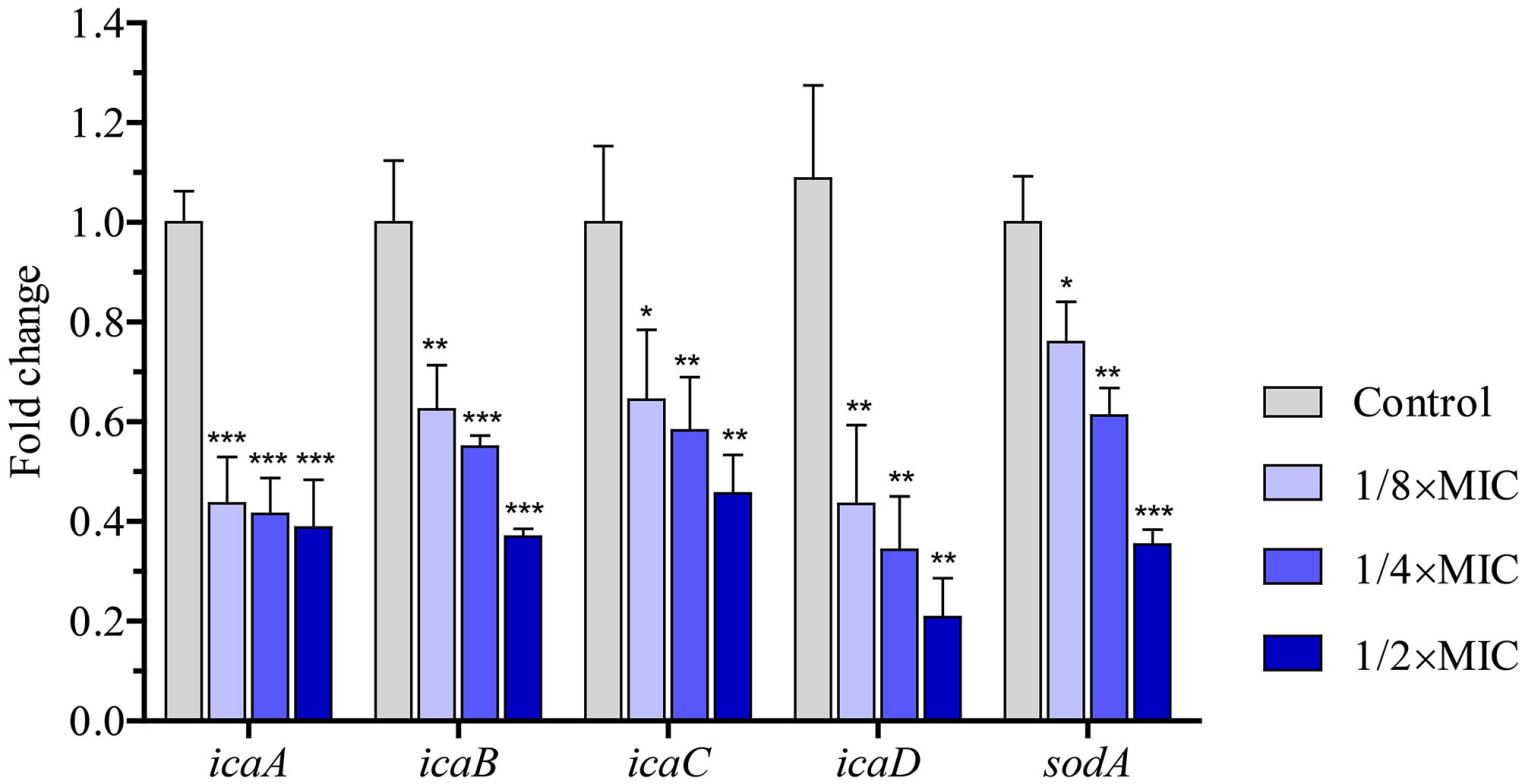
The EEE regulated the expression patterns of biofilm and ROS-associated genes. Quantification of the relative mRNA levels of *icaA*, *icaB*, *icaC*, *icaD*, and *sodA* in *S. aureus* cells treated with DMSO (control), 1/8×MIC, 1/4×MIC, and 1/2×MIC of the extract for 4 h. The fold changes in mRNA levels were indicated relative to *ftsZ* and control levels. Each data bar was presented as mean ± SD of three independent experiments. Statistical differences between control and EEE-treated samples were calculated by Student’s *t*-test (*, *p* ≤ 0.05; **, *p* ≤ 0.01; ***, *p* ≤ 0.001).

Our aforementioned results demonstrated the activity of the EEE on biofilm and ROS content (Fig. 4). To gain insight into the extract’s molecular mechanism of action, we examined the expression of *icaABCD* operon genes, which mediate the biosynthesis of polysaccharide intercellular adhesin, a molecule of bacterial biofilm, and *sodA*, which encodes a superoxide radical-converting enzyme, a negative regulator of ROS production [32, 33]. qRT-PCR analysis showed that the mRNA levels of all experimental genes were significantly downregulated upon the extract treatment (Fig. 5). These changes could be responsible for the decrease of biofilm formation and the increase of ROS level when bacterial cells were exposed to the extract.

## Discussion

Qualitative tests in this study revealed that the contents of phenolics, terpenoids, and alkaloids were insignificant in the EEE. In a previous study, several terpenoids, including bakuchiol, curculigosaponin A, dehydrolindestrenolide, and 1-methyl-3-(1-methyl-ethyl)-benzene, were identified in a methanol extract of Indonesian EBN [34]. In another study, the quantification of total phenolic content (TPC) by Folin-Ciolcalteu method showed high but variable TPCs (2.79 to 19.29 mg GAE/g) in Malaysian EBN samples extracted by water [35]. The differences in biochemical composition between our extract and the extracts by Permatasari *et al*. and Quek *et al*. might have resulted from variations in extraction method, production origin, species origin, or geographical origin.

On the contrary, proteins are present in the EEE and peptides make up the majority of the extract composition. This might be explained by the fact that some proteins and peptides in EBN can be solubilized and ultimately extracted by ethanol solvent. The solubility of proteins and peptides in ethanol was also observed in the studies of the leaf bean extract and the hydrolysates of sturgeon (*Acipenser schrenckii*) cartilage [36, 37]. Since approximately 37 proteins and 26 peptides of EBN have been described in recent studies, it is necessary to examine which proteins and peptides are present in the EEE and isolate potential antimicrobial agents in further studies [38, 39].

Our study was the first to demonstrate the potent antibacterial activity of a peptide-rich extract of EBN and its multiple modes of action on *S. aureus*. Although the inhibitory effect on *S. aureus* was also described for natural peptide-rich extracts from *Calliandra portoricensis* and the marine mollusk *Olivancillaria hiatula*, comprehensive mechanisms have yet to be elucidated [40, 41]. In the present work, the EEE was shown to exert activities on the bacterial cell membrane, biofilm, and ROS. In one route, the EEE disrupted the cell membrane integrity and potential of *S. aureus*. Furthermore, the extract inhibited biofilm formation via downregulating the biosynthesis genes of polysaccharide intercellular adhesin (*icaA*, *icaB*, *icaC*, and *icaD*). Elevation of ROS content by reducing the expression of an ROS inhibitor (*sodA*), which might result in oxidative cell damage and ultimately cell death, is another route of action of the EEE. As the destruction of cell membrane permeability can lead to profound changes in cell membrane integrity, further investigations on how the EEE interacts with the bacterial cell membrane and impacts its permeability should be performed [27].

*S. aureus* is a notorious, gram-positive bacteria pathogen involved in a wide range of mortal infections. This bacterium is also able to rapidly acquire resistance to various antibiotic drugs, while the emergence of the renowned methicillin-resistant *S. aureus* (MRSA) in particular has challenged the utility of traditional antibiotics [13]. However, *S. aureus* has also spurred explorations of new weapons against it in which antimicrobial peptides hold great promise to combat this species [42]. The antibacterial activities of our peptide-rich extract against *S. aureus* highlight it as an encouraging candidate for the treatment of bacterial infections. Moreover, the effect of the extract on MRSA strains could be an interesting project needing evaluation in further work.

## Supplemental Materials

Supplementary data for this paper are available on-line only at http://jmb.or.kr.



## References

[1] Kathan RH, Weeks DI (1969). Structure studies of collocalia mucoid. I. Carbohydrate and amino acid composition. Arch. Biochem. Biophys..

[2] Marcone MF (2005). Characterization of the edible bird's nest the 'Caviar of the East'. Food Res. Int..

[3] Chok KC, Ng MG, Ng KY, Koh RY, Tiong YL, Chye SM (2021). Edible bird's nest: recent updates and industry insights based on laboratory findings. Front. Pharmacol..

[4] Fan Q, Lian J, Liu X, Zou F, Wang X, Chen M (2021). A study on the skin whitening activity of digesta from edible bird's nest: a mucin glycoprotein. Gels.

[5] Guo CT, Takahashi T, Bukawa W, Takahashi N, Yagi H, Kato K (2006). Edible bird's nest extract inhibits influenza virus infection. Antiviral. Res..

[6] Bai W, Deng F, Zhang X, Han Y, Xiao Ye, Wang N (2023). The determination of epidermal growth factor in Edible bird's nest by enzyme-linked immunosorbent assay. Appl. Biol. Chem..

[7] Hou Z, Imam MU, Ismail M, Azmi NH, Ismail N, Ideris A (2015). Lactoferrin and ovotransferrin contribute toward antioxidative effects of edible bird's nest against hydrogen peroxide-induced oxidative stress in human SH-SY5Y cells. Biosci. Biotechnol. Biochem..

[8] Babji A, Syarmila EI, D'Aliah N, Nadia NM, Akbar HD, Norrakiah A (2018). Assessment on bioactive components of hydrolysed edible bird nest. Int. Food Res. J..

[9] Hun LT, Wani WA, Tjih ETT, Adnan NA, Le Ling Y, Aziz RA (2015). Investigations into the physicochemical, biochemical and antibacterial properties of edible bird's nest. Chem. Pharm. Res..

[10] Saengkrajang W, Matan N (2011). Antimicrobial activities of the edible bird's nest extracts against food-borne pathogens. Thai J. Agric. Sci..

[11] Chen W, Hwang YY, Gleaton JW, Titus JK, Hamlin N (2018). Optimization of a peptide extraction and LC-MS protocol for quantitative analysis of antimicrobial peptides. Future Sci. OA.

[12] Plaskova A, Mlcek J (2023). New insights of the application of water or ethanol-water plant extract rich in active compounds in food. Front. Nutr..

[13] Cheung GY, Bae JS, Otto M (2021). Pathogenicity and virulence of *Staphylococcus aureus*. Virulence.

[14] Prakobdi C, Saetear P (2023). Iodoform reaction-based turbidimetry for analysis of alcohols in hand sanitizers. Analytica.

[15] Bradford MM (1976). A rapid and sensitive method for the quantitation of microgram quantities of protein utilizing the principle of protein-dye binding. Anal. Biochem..

[16] Das B, Al-Amin M, Russel S, Kabir S, Bhattacherjee R, Hannan J (2014). Phytochemical screening and evaluation of analgesic activity of *Oroxylum indicum*. Indian J. Pharm. Sci..

[17] Kancherla N, Dhakshinamoothi A, Chitra K, Komaram RB (2019). Preliminary analysis of phytoconstituents and evaluation of anthelminthic property of *Cayratia auriculata* (in vitro). Maedica.

[18] Sreevidya N, Mehrotra S (2003). Spectrophotometric method for estimation of alkaloids precipitable with Dragendorff 's reagent in plant materials. J. AOAC Int..

[19] Nguyen TP, Vu Thi NA, Nguyen Diep XN, Nguyen T, Bui L (2022). Antimicrobial resistance tendency and collateral sensitivity of *Staphylococcus aureus* adapted to antibiotics or extracts of medicinal plants grown in Vietnam. Lett. Appl. Microbiol..

[20] Zhang LL, Zhang LF, Xu JG (2020). Chemical composition, antibacterial activity and action mechanism of different extracts from hawthorn (*Crataegus pinnatifida Bge*.). Sci. Rep..

[21] Wan Y, Wang X, Zhang P, Zhang M, Kou M, Shi C (2021). Control of foodborne *Staphylococcus aureus* by shikonin, a natural extract. Foods.

[22] Wen Z, Zhao Y, Gong Z, Tang Y, Xiong Y, Chen J (2022). The mechanism of action of Ginkgolic acid (15: 1) against gram-positive bacteria involves cross talk with iron homeostasis. Microbiol. Spectr..

[23] Ninganagouda S, Rathod V, Singh D, Hiremath J, Singh AK, Mathew J (2014). Growth kinetics and mechanistic action of reactive oxygen species released by silver nanoparticles from *Aspergillus niger* on *Escherichia coli*. Biomed Res. Int..

[24] Schmittgen TD, Livak KJ (2008). Analyzing real-time PCR data by the comparative CT method. Nat. Protoc..

[25] Reeks BY, Champlin FR, Paulsen DB, Scruggs DW, Lawrence ML (2005). Effects of sub-minimum inhibitory concentration antibiotic levels and temperature on growth kinetics and outer membrane protein expression in *Mannheimia haemolytica* and *Haemophilus somnus*. Can. J. Vet. Res..

[26] Yousefpour Z, Davarzani F, Owlia P (2021). Evaluating of the effects of sub-MIC concentrations of gentamicin on biofilm formation in clinical isolates of *Pseudomonas aeruginosa*. Iran. J. Pathol..

[27] Li X, Zuo S, Wang B, Zhang K, Wang Y (2022). Antimicrobial mechanisms and clinical application prospects of antimicrobial peptides. Molecules.

[28] Clementi EA, Marks LR, Roche-Håkansson H, Håkansson A (2014). Monitoring changes in membrane polarity, membrane integrity, and intracellular ion concentrations in *Streptococcus pneumoniae* using fluorescent dyes. J. Vis. Exp..

[29] Jepras RI, Paul FE, Pearson SC, Wilkinson MJ (1997). Rapid assessment of antibiotic effects on *Escherichia coli* by bis-(1, 3-dibutylbarbituric acid) trimethine oxonol and flow cytometry. Antimicrob. Agents Chemother..

[30] Muhammad MH, Idris AL, Fan X, Guo Y, Yu Y, Jin X (2020). Beyond risk: bacterial biofilms and their regulating approaches. Front. Microbiol..

[31] Vaishampayan A, Grohmann E (2021). Antimicrobials functioning through ros-mediated mechanisms: current insights. Microorganisms.

[32] Idrees M, Sawant S, Karodia N, Rahman AJIJoER, Health P (2021). *Staphylococcus aureus* biofilm: morphology, genetics, pathogenesis and treatment strategies. Int. J. Environ. Res. Public Health..

[33] Karavolos MH, Horsburgh MJ, Ingham E, Foster S (2003). Role and regulation of the superoxide dismutases of *Staphylococcus aureus*. Microbiology.

[34] Permatasari HK, Permatasari QI, Taslim NA, Subali D, Kurniawan R, Surya R (2023). Revealing edible bird nest as novel functional foods in combating metabolic syndrome: comprehensive in silico, in vitro, and in vivo studies. Nutrients.

[35] Quek MC, Chin NL, Yusof YA, Law CL, Tan SW (2018). Characterization of edible bird's nest of different production, species and geographical origins using nutritional composition, physicochemical properties and antioxidant activities. Food Res. Int..

[36] Racusen D, Foote M (1963). Solubility of bean leaf protein in ethanol. Nature.

[37] Wang B, Li ZR, Chi CF, Zhang QH, Luo HY (2012). Preparation and evaluation of antioxidant peptides from ethanol-soluble proteins hydrolysate of *Sphyrna lewini* muscle. Peptides.

[38] Wang X, Hu D, Liao F, Chen S, Meng Y, Dai J (2023). Comparative proteomic analysis of edible bird's nest from different origins. Sci. Rep..

[39] Wu WJ, Li LF, Cheng HY, Fung HY, Kong HY, Wong TL (2022). Qualitative and quantitative analysis of edible bird's nest based on peptide markers by LC-QTOF-MS/MS. Molecules.

[40] Gasu EN, Ahor HS, Borquaye LS (2018). Peptide extract from *Olivancillaria hiatula* exhibits broad-spectrum antibacterial activity. Biomed Res. Int..

[41] Ogbole OO, Ndabai NC, Akinleye TE, Attah AFJBcm, therapies (2020). Evaluation of peptide-rich root extracts of *Calliandria portoriscensis* (Jacq.) Benth (Mimosaceae) for in vitro antimicrobial activity and brine shrimp lethality. BMC Complement. Med. Ther..

[42] Mohammad H, Thangamani S, N Seleem M (2015). Antimicrobial peptides and peptidomimetics-potent therapeutic allies for staphylococcal infections. Curr. Pharm. Des..

