# Supplementary figures and images for: Uncovering the Antibacterial Potential of a Peptide-Rich Extract of Edible Bird’s Nest against Staphylococcus aureus
Source: J Microbiol Biotechnol. 2024 Jul 12;34(8):1680–7. doi: 10.4014/jmb.2402.02052 (PMC11380515; doi:10.4014/jmb.2402.02052)

## Supplementary Figure

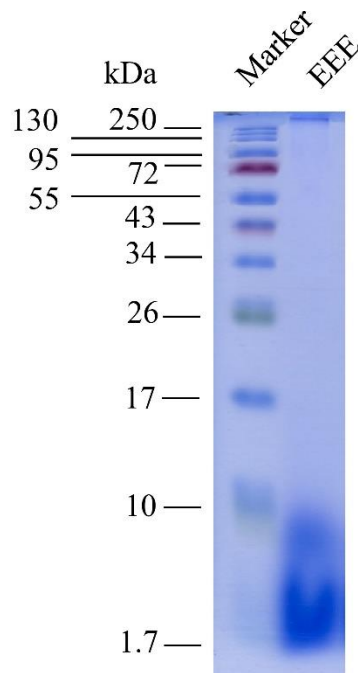

**Fig. S1. SDS-PAGE electropherogram of proteins and peptides in the EEE.**

Supplement: Supplementary file 1 [file jmb-34-8-1680-supple.pdf]
